# Anisodamine Maintains the Stability of Intervertebral Disc Tissue by Inhibiting the Senescence of Nucleus Pulposus Cells and Degradation of Extracellular Matrix via Interleukin-6/Janus Kinases/Signal Transducer and Activator of Transcription 3 Pathway

**DOI:** 10.3389/fphar.2020.519172

**Published:** 2020-12-15

**Authors:** Ning Tang, Yulei Dong, Chong Chen, Hong Zhao

**Affiliations:** Department of Orthopedic, Chinese Academy of Medical Sciences Peking Union Medical College Hospital, Beijing, China

**Keywords:** anisodamine, intervertebral disc degeneration, senescence, extracellular matrix, interleukin-6/janus kinases/signal transducer and activator of transcription 3 pathway

## Abstract

**Objectives:** Anisodamine (ANI) has been used to treat a variety of diseases. However, the study of ANI in intervertebral disc degeneration (IVDD) is unclear. This study investigated the effects of ANI on degenerative nucleus pulposus cells (NPCs) and IVDD rats, and its possible mechanisms.

**Methods:** Human nucleus pulposus cells (HNPCs) were treated with IL-1β (20 ng/ml) to simulate IVDD, and an IVDD rat model was constructed. IL-1β-induced HNPCs were treated with different concentrations (10, 20, or 40 μM) of ANI, and IVDD rats were also treated with ANI (1 mg/kg).

**Results:** ANI treatment significantly reduced the apoptosis, caspase-3 and SA-β-gal activities, and p53 and p21 proteins expression, while promoted telomerase activity and aggrecan and collagen II synthesis in IL-1β-induced HNPCs. Moreover, the introduction of ANI inhibited the expression of IL-6, phosphorylation of JAK and STAT3, and nuclear translocation of p-STAT3 in Degenerated HNPCs. Additionally, the application of ANI abolished the effects of IL-6 on apoptosis, SA-β-gal and telomerase activity, and the expression of p53, p21, aggrecan and collagen II proteins in degenerated HNPCs. Simultaneously, ANI treatment enhanced the effects of AG490 (inhibitor of JAK/STAT3 pathway) on IL-1β-induced apoptosis, senescence and ECM degradation in HNPCs. Furthermore, ANI treatment markedly inhibited the apoptosis and senescence in the nucleus pulposus of IVDD rats, while promoted the synthesis of aggrecan and collagen II. ANI treatment obviously inhibited JAK and STAT3 phosphorylation and inhibited nuclear translocation of p-STAT3 in IVDD rats.

**Conclusion:** ANI inhibited the senescence and ECM degradation of NPCs by regulating the IL-6/JAK/STAT3 pathway to improve the function of NPCs in IVDD, which may provide new ideas for the treatment of IVDD.

## Introduction

Intervertebral disc degeneration (IVDD) is the most common cause of lower back pain and is the basis of spinal degenerative diseases (Deng et al., 2017a). Due to the limited tissue regeneration ability of the lumbar intervertebral disc, it is difficult to be reversed after degeneration. Nucleus pulposus cells (NPCs) are the only constituent cells of the nucleus pulposus of intervertebral disc that the abnormal function of NPCs seriously affects the occurrence and development of IVDD (Rosenzweig et al., 2017). The main manifestations of IVDD are a decrease in the number of NPCs and a decrease in the synthesis of extracellular matrix (ECM). It is currently believed that IVDD is a pathological process involving multiple factors, which is related to genetic susceptibility, mechanical load, inflammatory cytokines, extracellular matrix degradation, cellular senescence and apoptosis.

Since IVDD is age-related, the senescence of various stress-induced NPCs plays a crucial role in the progression of IVDD (Gao et al., 2018). Senescence limits the division of NPCs and ultimately leads to apoptosis. Besides, senescence cells can also produce a large number of matrix degrading enzymes (MMPs) and inflammatory factors, further worsening the living environment of cells, which is also the pathological basis of IVDD. There is increasing evidence that signaling pathways play an important role in regulating the onset and persistence of senescence (Wang et al., 2006; Zirkel et al., 2018). After cellular senescence, a variety of kinases and transcription factors are activated, a process involving a variety of intracellular signal transduction pathways.

STAT3 is an important signal transduction molecule in cells that plays an important role in cell survival, apoptosis and senescence (Chipuk et al., 2010). STAT3 can be phosphorylated by a variety of kinases to form homologs or form heterodimers with other members of the STAT family, which in turn enter the nucleus from the cytoplasm to initiate transcription of downstream genes. The kinase that phosphorylates STAT3 includes JAK, proto-oncogene tyrosine-protein kinase, and the like. JAK/STAT3 can be activated by a variety of cytokines (such as IL-6) and growth factors (such as EGF). IL-6/JAK/STAT3 signaling pathway is the most important signaling pathway and its abnormal activation is related to various pathophysiological processes including inflammation, apoptosis and senescence (Liu et al., 2014; Johnson et al., 2018). As a multifunctional cytokine, IL-6 promotes the accumulation of inflammatory cells and stimulates the release of inflammatory mediators, aggravating the inflammatory response of IVDD (Deng et al., 2016). Additionally, IL-6 can directly participate in the regulation of intervertebral disc cell proliferation, apoptosis and ECM synthesis and decomposition imbalance, which in turn causes IVDD (Zhou et al., 2017). Moreover, IL-6 binds to its receptor and induces cellular senescence by activating the JAK/STAT3 pathway. Targeting silencing of the IL-6/STAT3 pathway in human intervertebral disc NPCs can delay the development of IDD by inhibiting ECM degradation to inhibit MMP-2 production (Ji et al., 2016).

Anisodamine (ANI), an alkaloid extracted from the root of *Anisodus tanguticus*, is an M-cholinergic receptor blocker. Studies have confirmed that ANI has the effect of relieving microvascular spasm and improving microcirculation (Poupko et al., 2007). ANI inhibits inflammatory response and apoptosis of renal tubular cells by reducing the expression of endoplasmic reticulum stress markers (IRE-1α, CHOP), NLRP3 inflammasome and inflammatory factors (IL-1α, IL-β and IL-18) (Yuan et al., 2017), and also reduces cardiomyocyte apoptosis by inhibiting oxidative stress, down-regulating the expression of caspase-3 and Bax (Yao et al., 2018). Furthermore, Luo and Zhou (2009) showed that ANI inhibits the deposition of ECM of experimental hepatic fibrosis by inhibiting MMP-2 expression. However, the role of ANI in IVDD is currently rarely studied.

As such, the present study was to investigate the effects of ANI on apoptosis, senescence and ECM degradation of degenerative human nucleus pulposus cells (HNPCs), and to explore its potential mechanism. Moreover, we also investigated the effect of ANI on IVDD *in vivo* by preparing a rat model of IVDD. We hope to provide new ideas for the treatment of IVDD.

## Materials and Methods

### Cell Culture

Human nucleus pulposus cells (HNPCs) were obtained from ScienCell Research Laboratories (Carlsbad, CA, United States) and cultured in Nucleus Pulposus Cell Medium (NPCM, ScienCell Research Laboratories) in a 37°C, 5% CO_2_ incubator. The NPCM was changed every 3 days. When fused to 80%, the cells were trypsinized and subcultured at a ratio of 3:1.

### Experimental Design

HNPCs were treated with 20 ng/ml of IL-1β for 48 h to induce a degenerated NPCs model (Deng et al., 2017b). The dose of ANI was determined by treating HNPCs with ANI (0–200 μM). Subsequently, cells were divided into four groups to analyze the effects of ANI on the apoptosis, senescence and ECM degradation of HNPCs: control group, IL-1β group, IL-1β+ANI-10 group, IL-1β+ANI-20 group and IL-1β+ANI-40 group. Moreover, rescue experiments were also performed by dividing the cells into five groups: control group, IL-1β group, IL-1β+ANI-40 group, IL-1β+IL-6 group, and IL-1β+IL-6+ANI-40 group. All treatments were performed for 48 h. Among them, the IL-1β+ANI + IL-6 group was first treated with IL-6 (10 mg/L) for 12 h, while IL-61β+ANI + AG490 group was first treated with AG490 (10 µM) for 12 h; then 40 μM ANI was added for the remaining time of 48 h.

### 3-(4,5-Dimethylthiazol-2-yl)-2,5-diphenyltetrazolium Bromide (MTT) Assay

HPNCs cells were seeded into 96-well plates (1 × 10^6^ cells/well), incubated overnight at 37°C, 5%CO_2_ incubator. Then, HPNCs were exposed to IL-1β (0–100 ng/ml) or ANI (0–200 μM) for 48 h. After treatments, cells were washed with fresh medium and added to MTT solution (Sigma-Aldrich, United States) and incubated for 4 h at room temperature. After aspirating the MTT solution, the cells were incubated with 100 μl DMSO for 5 min at room temperature. The optical density values of the plate were measured at 490 nm on a Tecan Sunrise Absorbance Microplate Reader (Tecan Group, Switzerland).

### Lactate Dehydrogenase (LDH) Assay

The cytotoxicity of ANI-treated HNPCs was evaluated by measuring the release of LDH using a CytoTox96 Non-Radioactive Cytotoxicity Assay kit (Beyotime, China). All procedures were performed in strict accordance with the kit instructions.

### Caspase-3 Activity

The activity of caspase-3 was measured using a caspase-3 activity assay kit (Beyotime, China). In brief, the treated HNPCs were incubated with 300 μl of lysis buffer for 15 min at 4°C. Subsequently, the supernatant was collected by centrifugation at 15,000 g for 15 min. 10 μl protein of cell lysate per sample in 80 μl reaction buffer were incubated for 4 h at 37°C and then placed on the auto microplate reader (Molecular Devices, Sunnyvale, CA, United States). The activity of caspase-3 was determined by measuring the absorbance at 405 nm.

### Senescence-Associated β-galactosidase Activity

Freshly prepared SA-β-gal staining fixative was added to the treated HNPCs according to the kit instructions (Beyotime, China) and incubated at 37°C for 3 h. After washing three times with 0.01 mol/L of PBS, the SA-β-gal staining solution was added dropwise, and placed in a humid box at 37°C overnight in the dark. The staining was observed under a light microscope. Five high power fields were randomly selected, and the senescence process of HNPCs was determined by calculating the ratio of SA-β-gal positive cells to the total number of cells (Wang et al., 2015).

### Telomerase Activity

After the treated HNPCs were lyzed and centrifuged, the supernatant was collected. Telomerase activity (IU/L) was measured using a telomerase enzyme-linked immunosorbent assay (ELISA) kit (MIbio, China) according to the manufacturer's instructions.

### Western Blot

Nuclear protein and total protein were extracted from IVDD rats' nucleus pulposus tissues and nucleus pulposus cells using Nuclear/Cytoplasmic protein Extraction Kit and Total protein Extraction Kit (AmyJet Scientific, Wuhan, China), respectively, according to the manufacturer’s instructions. Protein concentration was determined using the BCA method. The protein was separated by sodium dodecyl sulfate polyacrylamide gel electrophoresis and transferred to a polyvinylidene fluoride (PVDF) membrane. The membrane was blocked in Tris buffer containing 5% skim milk for 2 h. After washing with PBS, the membrane was incubated with anti-Bax antibody, anti-Bcl-2 antibody, anti-p53 antibody, anti-p21 antibody, anti-collagen II antibody, anti-aggrecan antibody, anti-IL-6 antibody, anti-JAK antibody, anti-Phospho-JAK antibody, anti-STAT3 antibody, anti-Phospho-STAT3 (Tyr-705) antibody, anti-Lamin B antibody, or anti-GAPDH antibody overnight at 4°C. After washing with PBS, the membrane was incubated with secondary antibody (1:1,000; Abcam) for 2 h at room temperature. Development was carried out using an enhanced chemiluminescent reagent. Protein was detected with Image Acquisition using Image Quant LAS 4000 (GE Healthcare Life Sciences, Marlborough, MA, United States).

### Hoechst 33258 Staining

NPCs were seeded into 6-well plates embedded with aseptically treated coverslips and cells were cultured to 80% confluence. The medium in the 6-well plate was aspirated, and the medium containing different concentrations of ANI was added, and cultured in a 37°C, 5% CO_2_ incubator for 36 h. Subsequently, the liquid in the well was aspirated and rinsed three times with PBS.4% paraformaldehyde was added to the well plate for 20 min at room temperature, followed by rinsing three times with PBS. Hoechst 33258 working solution was added to the well plate and stained for 20 min in the dark at room temperature, and then rinsed three times with PBS. Finally, the slide was sealed with an anti-fluorescence quenching solution (glycerol: PBS = 1:9), and observed under a fluorescence microscope and photographed.

### Immunofluorescence (IFC) Staining

NPCs were seeded into 24-well plates embedded with aseptically treated coverslips and cells were cultured to 80% confluence. The medium was removed and washed twice with PBS, and then the cells were fixed in 3.5% formaldehyde for 30 min at room temperature. After washing the cells three times with PBS, they were treated with 0.1% Triton X-100 in PBS for 20 min. Subsequently, the cells were incubated with 3% BSA and 0.05% Tween for 30 min at 37°C. Subsequently, the cells were incubated with rabbit monoclonal anti-p-STAT3 (1:1,000; Abcam) overnight at 4°C. After washing, cells were treated with fluorescent anti-rabbit secondary antibody (1:500; Abcam) for 2 h at room temperature. The nuclei were treated with 4,6-diamidino-2-phenylindole (DAPI). Fluorescence images were acquired under a co-aggregation microscope (Leica, Mannheim, Germany).

### Animal Model and Treatment

Male Sprague-Dawley rats (6 weeks) were provided by Institute of Life Sciences (Beijing, China). Rats were housed in standard cages with an ambient temperature of 23 ± 2°C, a humidity of 55% ± 10%, and a light and dark period of 12 h. The experiment was carried out after 1 week of adaptive feeding in rats. The IVDD rat model was prepared by fiber loop puncture (Jeong et al., 2010). In brief, the rat tail disc (Co4-5) was located on the coccygeal vertebrae. A 26G puncture needle was used to pierce the entire annulus of the fiber through the skin of the tail. The needle was held in the disc for 1 min. Immediate after the operation, ANI was intraperitoneally injected at a dose of 1 mg/kg per day (Ge et al., 2018). Rats in the control group and the IVDD group were injected with the same dose of physiological saline. Four weeks after surgery, rats were sacrificed by overdosing 0.1% sodium pentobarbital, tails were harvested and Co4-5 intervertebral disc samples were collected. All surgical interventions, treatments and postoperative animal care procedures were performed in strict accordance with the Animal Care and Use Committee of Peking Union Medical College Hospital.

### Immunochemistry

Immunohistochemistry was used to analyze the expression of p53, collagen II and p-STAT3 (phospho Y705) protein in rat tail intervertebral disc samples. After the sample was fixed with 4% paraformaldehyde for 24 h, decalcification, dehydration, waxing, and embedding treatment were performed to prepare paraffin sections (6 μm). Paraffin sections were dewaxed, hydrated, and incubated in freshly prepared 3% H_2_O_2_ for 10 min at room temperature. After the sections were repaired by microwave antigen for 20 min, goat serum was added and blocked at room temperature for 10 min. Sections were incubated with p53, collagen II and p-STAT3 (phospho Y705) antibody overnight at 4°C. After washing, biotinylated secondary antibodies were added to the sections for 4 h. Subsequently, freshly prepared DAB chromogenic solution was added to the sections for color development, and was terminated at the appropriate time under the microscope. The samples were counterstained with hematoxylin dye solution and rinsed with distilled water. After dehydration by gradient ethanol, the samples were sealed with a neutral gum. Five areas were selected under an optical microscope for photographing and preservation.

### Terminal Deoxynucleotidyl Transferase dUTP Nick-End Labeling (TUNEL) Assay

Frozen sections (5 μm) were prepared by taking 4% paraformaldehyde-fixed nucleus pulposus tissue and dehydrating in 20% sucrose overnight. Subsequently, the tail disc samples were subjected to an *in situ* TUNEL reaction using an ApopTag InSitu apoptosis detection kit (Millipore, Billerica, United States) according to the manufacturer’s protocol. Apoptotic cells were imaged under a light microscope (magnification, ×400). Among them, red fluorescence was used to label apoptotic nucleus pulposus nuclei, and blue DAPI was used to label all nuclei. Red and blue were superimposed on apoptotic cells.

### Statistical Analysis

All statistical analyses were performed using SPSS 20.0 software (IBM Corp., Armonk, NY, United States), and graphs were generated using GraphPad Prism 5 Software (Graph Pad Software, Inc., La Jolla, CA, United States). Student’s t test was used to analyze proteins expression. An ANOVA was also performed comparing more than two groups. *p* (two-tailed) <0.05 were considered statistically significant.

## Results

### Cytotoxicity Test

**FIGURE 1:**
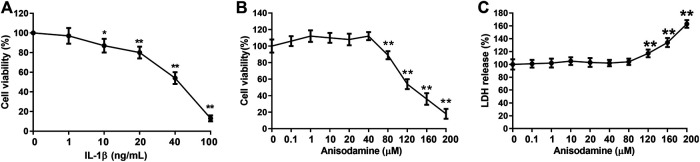
Cytotoxicity experiments. **(A)** The effect of IL-1β on the activity of HNPCs was analyzed by MTT assay. **(B)** The effect of ANI on the activity of HNPCs was analyzed by MTT assay. **(C)** The toxic effects of ANI on HNPCs were analyzed by LDH assay. ^*^ *p* ＜ 0.05, **p ＜ 0.01.

IL-1β can be used to establish IVDD *in vitro* (Deng et al., 2017). In order to study the effect of ANI on IVDD, IL-1β was used to treat HNPCs to induce IVDD *in vitro*. We found that when the concentration of IL-1β was greater than 10 ng/ml, the activity of HNPCs was delayed, and the inhibition was enhanced with increasing concentration (Figure 1A). Hence, we used 20 ng/ml of IL-1β for subsequent experiments. Additionally, the cytotoxicity of ANI to HNPCs was examined by treating HNPCs with different concentrations (0–200 μM) of ANI for 48 h. We found that ANI did not promote or inhibit the proliferation of HNPCs at concentrations ranging from 0 to 40 μM (Figure 1B). However, when the concentration was higher than 80 μM, the viability of cells was obviously decreased (Figure 1B), and cytotoxicity was also detected by the LDH assay (Figure 1C). Therefore, we selected ANI at concentrations of 10, 20, and 40 μM for subsequent experiments.

#### ANI Inhibited Apoptosis, Senescence and ECM Degradation of HNPCs Induced by IL-1β

**FIGURE 2:**
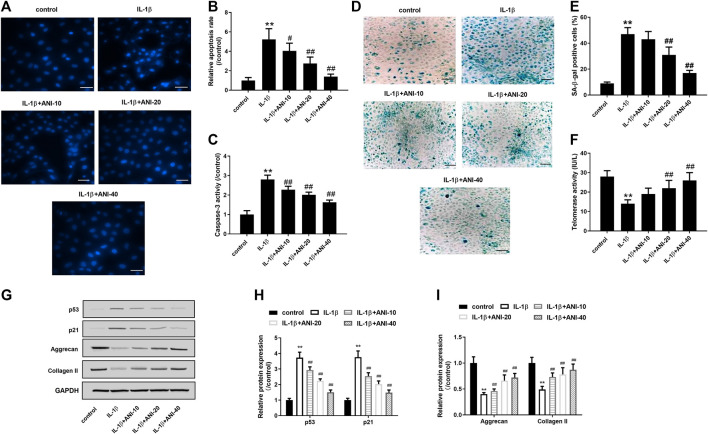
ANI inhibited apoptosis, senescence and ECM degradation of HNPCs induced by IL-1β. **(A,B)** Hoechst 33258 staining was used to analysis the apoptosis of HNPCs. Bar = 50 μm. **(C)** The activity of caspase-3 in HNPCs was detected by a caspase-3 activity kit showed that ANI inhibited caspase-3 activity of HNPCs induced by IL-1β. **(D,E)** SA-β-gal staining was used to analyze senescence of HNPCs. Bar = 100 μm. **(F)** Telomerase activity assay was used to analyze senescence of HNPCs. **(G–I)** The expression of p53, p21, aggrecan and collagen II in HNPCs treated with or without ANI was analyzed by western blot assay. All experiments were performed as means ± SD of three times in duplicates. ^*^ *p* ＜ 0.05, **p ＜ 0.01 vs. control group; ^#^ *p* ＜ 0.05, ^##^ *p* ＜ 0.01 vs. IL-1β group.

Next, we used different concentrations (10, 20, 40 μM) of ANI to treat IL-1β-induced HNPCs to analyze the effect of ANI on IVDD *in vitro*. Hoechst33258 staining showed that the apoptosis of HNPCs after IL-1β treatment was obvious, and the apoptosis rate of IL-1β group was obviously higher than that of control group (*p* < 0.05), while ANI treatment could markedly reduce the apoptosis of HNPCs induced by IL-1β (*p* < 0.05, Figures 2A, B). Further analysis of the activity of the apoptotic initiation marker protein caspase-3 revealed that the activity of caspase-3 in the IL-1β group was evidently higher than that in the control group (*p* < 0.05), while ANI treatment inhibited the activation of caspase-3 in degenerating HNPCs in a dose-dependent manner (*p* < 0.05, Figure 2C).

Senescence associated β-galactosidase (SA-β-gal) activation is a hallmark feature of cellular senescence (Strzeszewska et al., 2018), and p53 is the most important senescence regulatory protein that promotes cellular senescence (Kim et al., 2009). A decrease in telomerase activity reflects an increase in cellular senescence (Li et al., 2019). In the present study, SA-β-gal staining showed that the proportion of cells stained with SA-β-gal positively in IL-1β-induced HNPCs was markedly increased, while treatment with 20 and 40 μM of ANI markedly reduced the proportion of SA-β-gal positive cells (Figures 2D,E). Telomerase activity assay further confirmed that ANI treatment significantly improved IL-1β-induced senescence of HNPCs (Figure 2F). What is more, western blot analysis showed that IL-1β could induce the increase of p53 and p21 protein expression in HNPCs, while ANI treatment could inhibit the expression of p53 and p21 proteins in degenerative HNPCs (*p* < 0.05, Figures 2G,H).

HNPCs mainly secrete aggrecan and collagen II, which are also the main components of ECM. We next observed the effect of ANI on the extracellular matrix. The results showed that collagen II and aggrecan proteins expression in IL-1β-induced HNPCs was significantly decreased, while ANI could promote the synthesis of collagen II and aggrecan (*p* < 0.05, Figures 2G,I). These results suggested that ANI may mitigate disc degeneration by reducing apoptosis, senescence and ECM degradation of HNPCs.

#### ANI Inhibited Activation of Interleukin-6/Janus Kinases/Signal Transducer and Activator of Transcription3 Pathway in Human nucleus pulposus cells Induced by IL-1β

**FIGURE 3:**
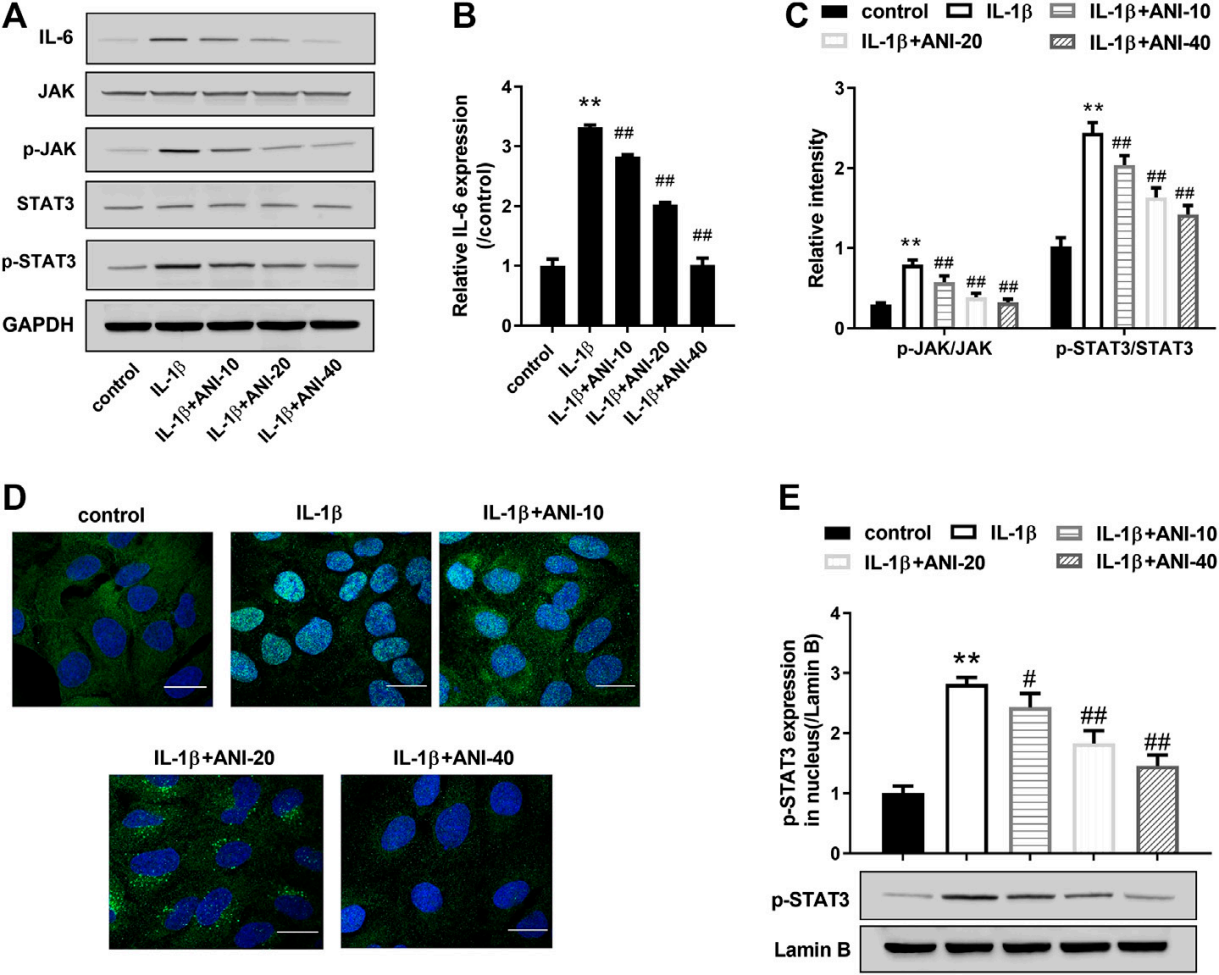
ANI inhibited IL-1β-induced activation of IL-6/JAK/STAT3 pathway in HNPCs. **(A–C)** The expression of IL-6, JAK, *p*-JAK, STAT3 and p-STAT3 in HNPCs was analyzed by western blot assay. **(D)** Immunofluorescence staining was used to analyze nuclear translocation of p-STAT3 in HNPCs. Bar = 25 μm. **(E)** The expression of p-STAT3 in nucleus was analyzed by western blot assay. ^*^ *p* ＜ 0.05, **p ＜ 0.01 vs. control group; ^#^ *p* ＜ 0.05 vs IL-1β group.

To investigate the possible mechanism by which ANI improves IVDD, we analyzed changes in IL-6/JAK/STAT3 signaling pathways in degenerative NPCs. Western blot analysis showed that IL-1β treatment significantly increased IL-6 expression in HNPCs and promoted phosphorylation of STAT3 and JAK compared with the control group (Figures 3A–C). Moreover, IGF staining also observed that IL-1β treatment induced nuclear translocation of p-STAT3 in HNPCs (Figure 3D), and increased the expression of p-STAT3 in the nucleus (Figure 3E). Compared with the IL-1β group, ANI treatment inhibited IL-6 expression, phosphorylation of JAK and STAT3, and nuclear translocation of p-STAT3 in HNPCs. These results suggested that ANI treatment could inhibit the activation of the IL-6/JAK/STAT3 pathway in IL-1β-induced HNPCs.

#### ANI Inhibited IL-1β-Induced Apoptosis, Senescence and ECM Degradation of HNPCs by Regulating IL-6/JAK/STAT3 Signaling Pathway

**FIGURE 4:**
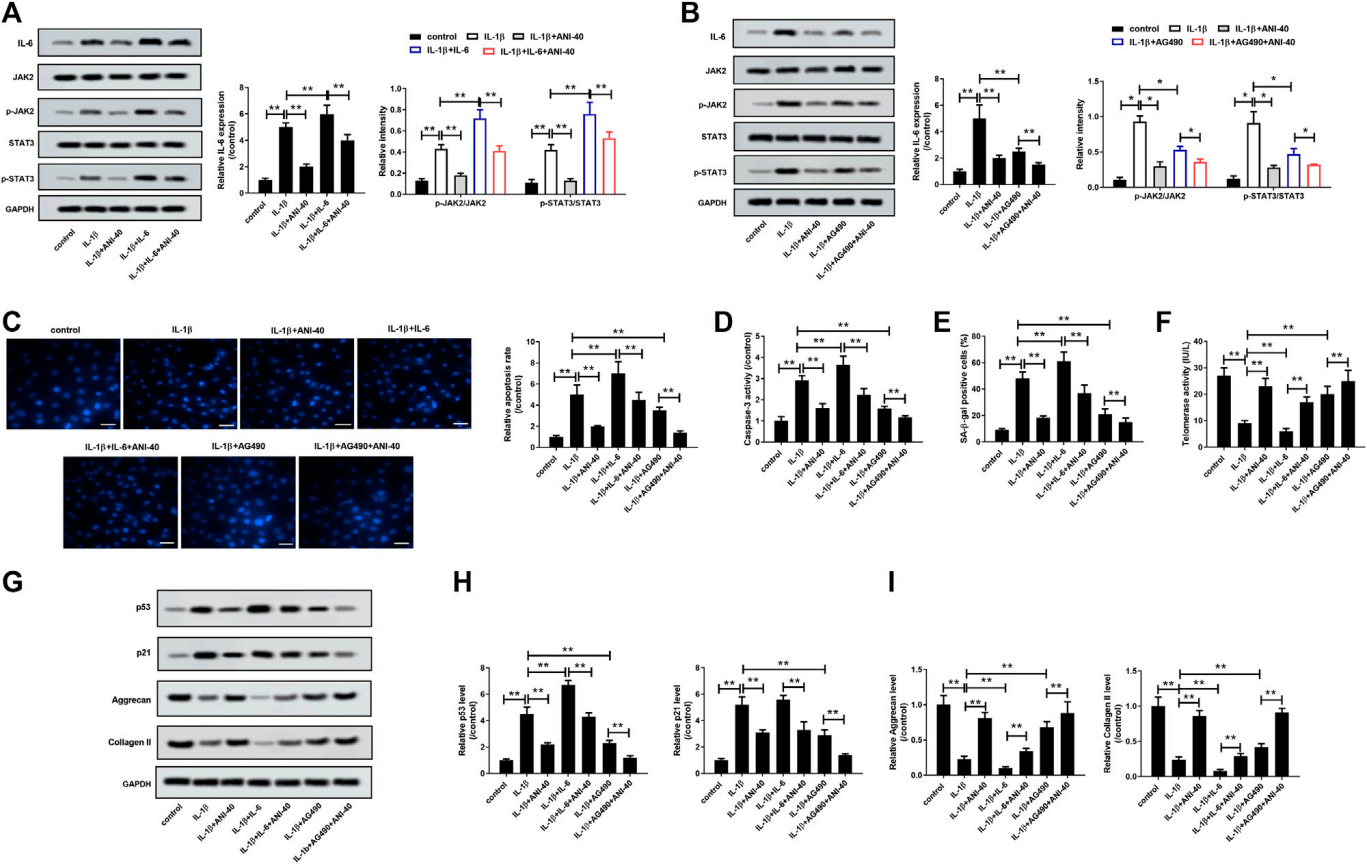
ANI inhibited IL-1β-induced apoptosis, senescence, and ECM degradation of HNPCs by modulating the IL-6/JAK/STAT3 signaling pathway. **(A,B)** The expression of IL6, JAK, *p*-JAK, STAT3 and p-STAT3 in HNPCs was analyzed by western blot assay. **(C)** Hoechst 33258 staining was used to analyze the apoptosis of HNPCs. Bar = 50 μm. **(D)** Detection of caspase-3 activity. SA-β-gal staining **(E)** and telomerase activity assay **(F)** were used to analyze senescence of HNPCs. Bar = 100 μm. **(G–I)** The expression of p53, p21, aggrecan and collagen II in HNPCs was analyzed by western blot assay. ^*^ *p* ＜ 0.05, **p ＜ 0.

To analyze whether ANI suppresses HNPCs degradation by inhibiting the IL-6/JAK/STAT3 signaling pathway, we performed a rescue experiment on IL-1β-induced HNPCs with IL-6 (10 mg/L) or PI3K/AKT inhibitor AG490 (10 µM) prior to treatment with ANI (40 μM). As expected, IL-6 treatment could enhance the activation of IL-1β stimulation on the IL-6/JAK/STAT3 pathway, which was reflected by increased IL-6 expression and increased phosphorylation levels of JAK and STAT3, while ANI treatment could eliminate this effect to a certain extent (Figure 4A). Moreover, IL-6 treatment could eliminate the activation of IL-6/JAK/STAT3 pathway stimulated by IL-1β, and ANI treatment enhanced this effect (Figure 4B). The results showed that IL-6 treatment could promote apoptosis (Figure 4C), caspase-3 activity (Figure 4D), SA-β-gal activity (Figure 4E), and p53 and p21 proteins expression (Figures 4G,H), while decreased telomerase activity (Figure 4F), and aggrecan and collagen II synthesis (Figures 4G,I). More importantly, the apoptotic rate and caspase-3 activity in the IL-1β+IL-6+ANI-40 group were significantly lower than those in the IL-1β+IL-6 group (Figures 4C,D). Furthermore, ANI abolished the effects of IL-6 on SA-β-gal activity (Figure 4C), telomerase activity (Figure 4F), and p53 and p21 proteins expression (Figures 4G,H). Moreover, ANI reversed the inhibitory effect of IL-6 on the synthesis of aggrecan and collagen II (Figures 4G,I) in IL-1β-induced HNPCs. Moreover, ANI treatment enhanced the effects of AG490 on IL-1β-induced apoptosis, senescence and ECM degradation in HNPCs (Figures 4C–I). These results revealed that ANI may play a protective role in degenerative HNPCs by regulating the IL-6/JAK/STAT3 pathway.

#### ANI Inhibited Apoptosis, Senescence and ECM Degradation of Nucleus Pulposus Tissue by Inhibiting IL-6/JAK/STAT3 Signaling Pathway *In Vivo*

**FIGURE 5:**
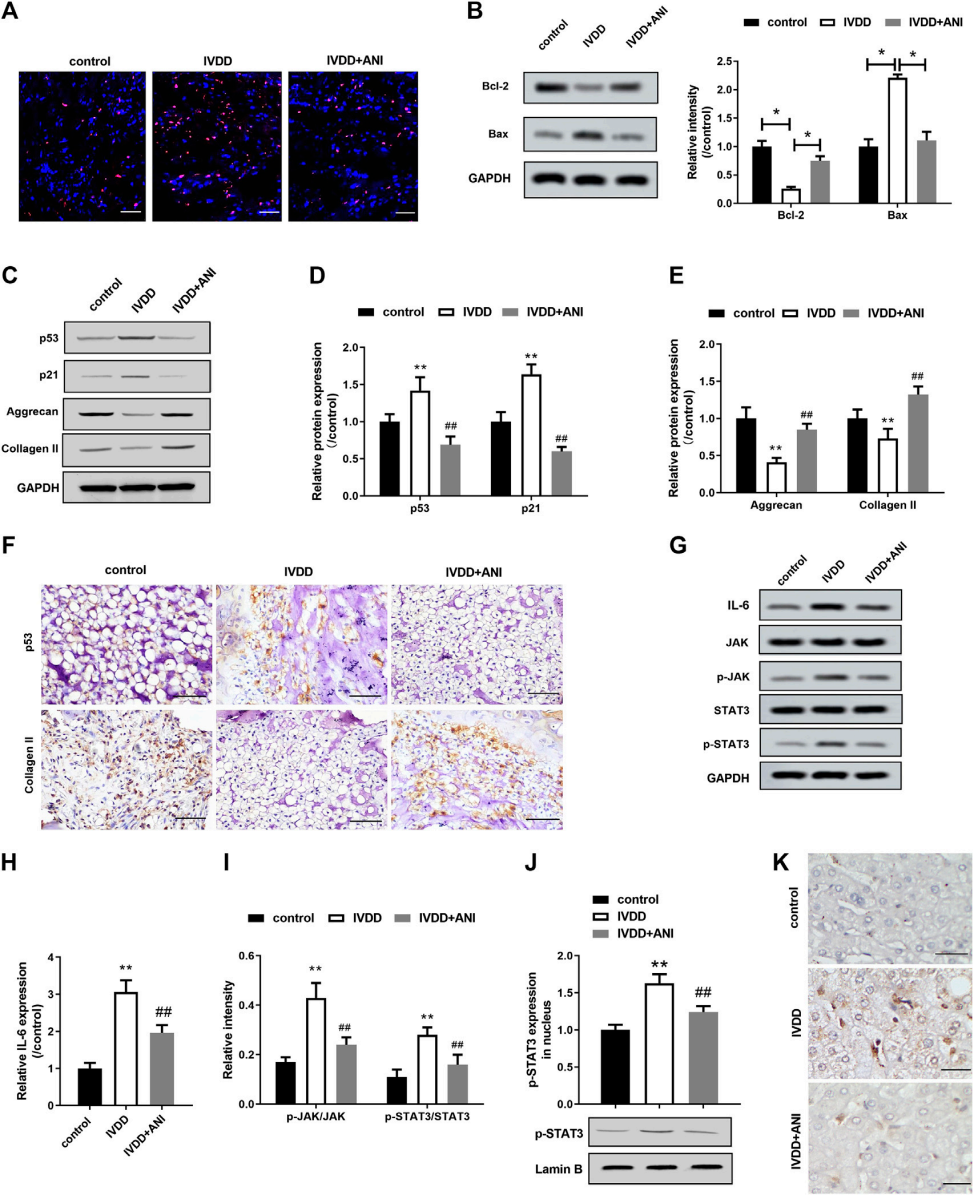
ANI inhibited apoptosis, senescence and ECM degradation in nucleus pulposus tissue by inhibiting IL-6/JAK/STAT3 signaling pathway. **(A)** TUNEL assay was used to analyze apoptosis in the nucleus pulposus tissue of IVDD rats with or without ANI treatment. Bar = 100 μm. Red was the apoptotic nucleus pulposus nucleus, and blue was the nucleus. **(B)** Western blot assay was used to analyze the expression of Bcl-2 and Bax proteins in the nucleus pulposus of IVDD rats with or without ANI treatment. **(C–E)** Western blot assay was used to analyze the expression of p53, p21, aggrecan and collagen II proteins in nucleus pulposus tissue of IVDD rats with or without ANI treatment. **(F)** Immunohistochemistry was used to analyze the expression of p53 and aggrecan proteins in nucleus pulposus tissues. Bar = 100 μm. **(G–I)** Western blot analysis was used to analyze the expression of *p*-JAK and p-STAT3 in the nucleus pulposus of IVDD rats with or without ANI treatment. **(J)** Western blot assay was used to analyze the expression of p-STAT3 protein in the nucleus pulposus tissue. **(K)** The localization of p-STAT3 in nucleus pulposus tissues was analyzed by immunohistochemistry. Bar = 50 μm. *N* = 6. ^*^ *p* ＜ 0.05 vs. control; ^#^ *p* ＜ 0.05 vs. IVDD.

We treated IVDD rats for 4 weeks by intraperitoneal injection of ANI to analyze the effect of ANI on IVDD *in vivo*. The TUNEL assay showed a significant decrease in apoptosis in the nucleus pulposus of the IVDD + ANI group (Figure 5A). Consistently, ANI increased Bcl-2 expression while decreased Bax expresson of IVDD rats (Figure 5B). Western blot analysis (Figures 5C,D) showed that ANI treatment markedly inhibited the expression of p53 and p21proteins in the nucleus pulposus of IVDD rats, whereas promoted the expression of aggrecan and collagen II (Figure 5E). Additionally, immunohistochemical analysis also showed that ANI treatment increased the expression of p53 and collagen II in the nucleus pulposus of IVDD rats (Figure 5F). What is more, ANI treatment significantly inhibited the expression of IL-6 expression, phosphorylation of STAT3 and JAK, and nuclear translocation of p-STAT3 in nucleus pulposus of IVDD rats (Figures 5G–J). Moreover, ANI treatment reduced the localization of p-STAT3 in the nucleus pulposus of IVDD rats (Figure 5K).

## Discussion

As an M receptor blocker, ANI has proven to be a relatively safe and effective drug that has been used in the treatment of various diseases including rheumatoid arthritis, ischemia-reperfusion injury, and respiratory diseases (Poupko et al., 2007; Zhou et al., 2014). However, the role of ANI in IVDD is unclear. This study found that ANI could inhibit IL-1β-induced apoptosis and senescence of HNPCs, and promote collagen II and synthesize *in vitro*. Consistent with *in vitro* study, ANI could improve the degeneration of intervertebral disc degeneration in IVDD rats by inhibiting the apoptosis and senescence of NPCs and inhibiting ECM degradation in the nucleus pulposus tissue. More importantly, we found that the improvement in IVDD by ANI may be related to its inhibition of IL-6/JAK/STAT3 signaling pathway.

Cellular senescence is a proliferative cell that continues to suffer from exogenous and endogenous stress and damage, entering a perpetual cell cycle arrest state (Yousefzadeh et al., 2019). Adult intervertebral discs are the largest avascular tissue in the body. Ischemia, hypoxia, and nutrient deprivation in the intervertebral disc increase the stress factors such as high lactate metabolism, high osmotic pressure, and oxidative damage, which induce premature senescence of NPCs (Feng et al., 2016). Apoptosis is the final link in the senescence of nucleus pulposus cells (Jung et al., 2019). We found that the apoptotic rate of degenerative HNPCs increased significantly, and the activity of caspase-3, a key executive molecule and the main effector molecule of apoptosis, was also significantly increased in IL-1β-induced HNPCs, suggesting the occurrence of apoptosis in degenerative HNPCs. SA-β-gal is a relatively specific cell senescence marker (Oh et al., 2018) that is derived from lysosome β-galactosidase that reflects increased expression of lysosomal β-galactosidase protein in senescent cells (Bo et al., 2006). Roberts et al. (2006) and Gruber et al. (2007) showed an increase in SA-β-gal staining-positive cells in degenerated intervertebral discs and nucleus pulposus compared with undegenerated discs. Two signaling pathways alone or synergistically induce cellular senescence: the p53/p21 and p16INK4A/pRB pathways, in which the p53/p21 pathway is dominant in IVDD (Kim et al., 2009). Progressive shortening of telomeres activates the expression of the tumor suppressor gene p53 by mimicking DNA damage signals, which in turn activates its downstream gene p21. Activation of p21 inhibits cyclin-dependent kinase two and cyclin E, which in turn reduces the degree of phosphorylation of retinoblastoma (RB) protein. The hypophosphorylated RB protein binds to and inactivates the nuclear transcription factor E2F, eventually causing senescence (Jeyapalan and Sedivy, 2008). Moreover, the p53 pathway can also be directly caused by cellular DNA damage induced by stress. Studies have shown that hyperosmolar states can activate this pathway by damaging the DNA of NPCs, and inactivation of p53 reverses this process (Mavrogonatou and Kletsas, 2009). We also observed an increase in activity of SA-β-gal, as well as p53 and p21 protein expression in IL-1β-induced HNPCs, and a decrease in telomerase activity, suggesting senescence in degenerative intervertebral discs. Senescence not only reduces the cell viability of NPCs, but also causes phenotypic changes that lead to the degradation of ECM (Gruber et al., 2007). ECM not only maintains the integrity of the intervertebral disc, but also regulates the survival, morphology and differentiation of NPCs by providing mechanical and biochemical pathways to NPCs. A typical feature of IVDD is the loss of ECM components. Aggrecan and collagen II are the most important components of nucleus pulposus ECM. We also observed a decrease in the expression of aggrecan and collagen II in IL-1β-induced HNPCs, suggesting degradation of ECM in HNPCs. These results demonstrated that IL-1β could induce degeneration of HNPCs *in vitro* by inducing apoptosis, senescence, and ECM degradation, thereby inducing IVDD. Consistent with *in vitro* studies, we also observed senescence, apoptosis, and ECM degradation in degenerated intervertebral disc nucleus tissues in IVDD rats.

Delaying the senescence of NPCs and promoting the synthesis of ECM components to inhibit the initiation of IVDD is expected to be the key to the treatment of IVDD (Cai et al., 2016; Chen et al., 2018). ANI has been shown to have anti-inflammatory, anti-apoptotic and anti-oxidative effects. The present study showed that ANI inhibited apoptosis and SA-β-gal activity, whereas increased telomere activity and collagen II and aggrecan synthesis in IL-1β-induced HNPCs. Simultaneously, the results also showed in the IVDD rat model that ANI treatment could increase Bcl-2 expression while reduce Bax expression in nucleus pulposus tissue, which are key regulators of apoptosis (Chipuk et al., 2010; Delbridge et al., 2016), inhibited the expression of p53 and p21, and promoted the synthesis of collagen II and aggrecan. Zhang et al. (2019) also showed that moxibustion treatment may be beneficial to IVDD by reducing apoptosis, which is manifested in the up-regulation of Bcl-2 expression and down-regulation of Bax expression. Collectively, these results revealed that ANI may extenuate IVDD by inhibiting apoptosis, senescence, and ECM degradation of NPCs.

As one of the pro-inflammatory cytokines, IL-6 has been shown to accelerate IVDD (Risbud and Shapiro, 2014). IL-6 forms a complex IL-6/IL6R/gp130 by binding to its receptor IL-6R, and dimerization of the gp130 molecule results in phosphorylation with JAK. Activated JAK further phosphorylates STAT3, which forms a dimer and translocates to the nucleus to activate transcription and expression of the corresponding target gene, such as Bcl-xL, Bcl-2, CyclinD1, Fas, VEGF, MMP-2, etc. (Wang and Sun, 2014). Blocking the IL-6/JAK/STAT3 pathway has become a new strategy for the treatment of disease (Johnson et al., 2018). For example, IL-6 and its receptor IL-6R monoclonal antibody have been used in the treatment of rheumatoid arthritis (Pelechas et al., 2017) and uveitis (Elkinson and McCormack, 2013), and JAK small molecule inhibitors have also been used in the treatment of rheumatoid arthritis and myelofibrosis (Mascarenhas and Hoffman, 2012; Traynor, 2012). We found that ANI inhibited the expression of IL-6 and the phosphorylation of JAK and STAT3 in IL-1β-induced HNPCs, and also inhibited nuclear translocation of p-STAT3 *in vitro*. Additionally, IL-6 reversed the inhibitory effect of ANI on IL-6/JAK/STAT3 pathway activation *in vitro*. And, IL-6 could eliminate the anti-apoptosis, anti-aging and anti-ECM degradation effects of ANI on degenerative HNPCs to some extent. More importantly, ANI abolished the effects of IL-6 on apoptosis, senescence and ECM degradation of degenerative HNPCs. Additionally, ANI treatment enhanced the effects of AG490 (inhibitor of JAK/STAT3 pathway) on IL-1β-induced apoptosis, senescence and ECM degradation in HNPCs. Moreover, ANI treatment was also observed in IVDD rats to inhibit the activation of the IL-6/JAK/STAT3 pathway in nucleus pulposus tissues. Kojima et al. (2012) found that IL-6 and soluble IL-6R stimulation induce cellular senescence by causing DNA damage response and p53 accumulation, a process that uses STAT3 as a major trigger and enhancer component. Inhibition of IL-6/JAK/STAT3 pathway activation can delay IVDD by inhibiting apoptosis of NPCs and degradation of ECM (Suzuki et al., 2017; Zhou et al., 2017). Based on these studies, we hypothesized that ANI may play a protective role in IVDD by inhibiting the activation of IL-6/JAK/STAT3 pathway to suppress apoptosis, senescence and ECM degradation of NPCs.

## Conclusion

Taken together, ANI could improve the function of NPCs in IVDD by inhibiting the apoptosis, senescence and ECM degradation via negatively regulating IL-6/JAK/STAT3 signaling pathway. This study may provide insight for further study into the protective effects of ANI. However, further studies are needed to demonstrate the effect of ANI in IVDD to promote the clinical treatment of IVDD.

## Data Availability Statement

All datasets generated for this study are included in the article/Supplementary Material.

## Ethics Statement

The animal study was reviewed and approved by Animal Care and Use Committee of Peking Union Medical College Hospital.

## Author Contributions

NT and HZ designed the research; NT and YD performed the research; CC analyzed the data and wrote the paper; and HZ conceived the idea and supervised the whole project. All authors discussed the results and commented on the manuscript.

## Conflict of Interest

The authors declare that the research was conducted in the absence of any commercial or financial relationships that could be construed as a potential conflict of interest.
